# Population-Based Impact of Smoking, Drinking, and Genetic Factors on HDL-cholesterol Levels in J-MICC Study Participants

**DOI:** 10.2188/jea.JE20210142

**Published:** 2023-04-05

**Authors:** Yora Nindita, Masahiro Nakatochi, Rie Ibusuki, Ippei Shimoshikiryo, Daisaku Nishimoto, Keiichi Shimatani, Toshiro Takezaki, Hiroaki Ikezaki, Masayuki Murata, Megumi Hara, Yuichiro Nishida, Takashi Tamura, Asahi Hishida, Mako Nagayoshi, Rieko Okada, Keitaro Matsuo, Hidemi Ito, Haruo Mikami, Yohko Nakamura, Takahiro Otani, Sadao Suzuki, Teruhide Koyama, Etsuko Ozaki, Kiyonori Kuriki, Naoyuki Takashima, Naoko Miyagawa, Kokichi Arisawa, Sakurako Katsuura-Kamano, Yukihide Momozawa, Michiaki Kubo, Kenji Takeuchi, Kenji Wakai

**Affiliations:** 1 Department of International Island and Community Medicine, Kagoshima University Graduate School of Medical and Dental Sciences, Kagoshima, Japan; 2 Department of Pharmacology and Therapeutic, Faculty of Medicine, Diponegoro University, Semarang, Indonesia; 3 Public Health Informatics Unit, Department of Integrated Health Sciences, Nagoya University Graduate School of Medicine, Nagoya, Japan; 4 School of Health Sciences, Faculty of Medicine, Kagoshima University, Kagoshima, Japan; 5 Division of Nursing, Higashigaoka Faculty of Nursing, Tokyo Healthcare University, Tokyo, Japan; 6 Department of Comprehensive General Internal Medicine, Kyushu University Graduate School of Medical Sciences, Faculty of Medical Sciences, Fukuoka, Japan; 7 Department of General Internal Medicine, Kyushu University Hospital, Fukuoka, Japan; 8 Department of Preventive Medicine, Faculty of Medicine, Saga University, Saga, Japan; 9 Department of Preventive Medicine, Nagoya University Graduate School of Medicine, Nagoya, Japan; 10 Division of Cancer Epidemiology and Prevention, Aichi Cancer Center Research Institute, Nagoya, Japan; 11 Division of Cancer Information and Control, Aichi Cancer Center Research Institute, Nagoya, Japan; 12 Cancer Prevention Center, Chiba Cancer Center Research Institute, Chiba, Japan; 13 Department of Public Health, Nagoya City University Graduate School of Medical Sciences, Nagoya, Japan; 14 Department of Epidemiology for Community Health and Medicine, Kyoto Prefectural University of Medicine, Kyoto, Japan; 15 Laboratory of Public Health, Division of Nutritional Sciences, School of Food and Nutritional Sciences, University of Shizuoka, Shizuoka, Japan; 16 Department of Public Health, Kindai University Faculty of Medicine, Osaka, Japan; 17 Department of Public Health, Shiga University of Medical Science, Otsu, Japan; 18 Department of Preventive Medicine and Public Health, Keio University School of Medicine, Tokyo, Japan; 19 Department of Preventive Medicine, Tokushima University Graduate School of Biomedical Sciences, Tokushima, Japan; 20 Laboratory for Genotyping Development, Center for Integrative Medical Sciences, RIKEN, Yokohama, Japan

**Keywords:** HDL-cholesterol, drinking, smoking, single nucleotide polymorphism, gene-environmental interaction

## Abstract

**Background:**

Environmental and genetic factors are suggested to exhibit factor-based association with HDL-cholesterol (HDL-C) levels. However, the population-based effects of environmental and genetic factors have not been compared clearly. We conducted a cross-sectional study using data from the Japan Multi-Institutional Collaborative Cohort (J-MICC) Study to evaluate the population-based impact of smoking, drinking, and genetic factors on low HDL-C.

**Methods:**

Data from 11,498 men and women aged 35–69 years were collected for a genome-wide association study (GWAS). Sixty-five HDL-C-related SNPs with genome-wide significance (*P* < 5 × 10^−8^) were selected from the GWAS catalog, of which seven representative SNPs were defined, and the population-based impact was estimated using population attributable fraction (PAF).

**Results:**

We found that smoking, drinking, daily activity, habitual exercise, egg intake, BMI, age, sex, and the SNPs *CETP* rs3764261, *APOA5* rs662799, *LIPC* rs1800588, *LPL* rs328, *ABCA1* rs2575876, *LIPG* rs3786247, and *APOE* rs429358 were associated with HDL-C levels. The gene-environmental interactions on smoking and drinking were not statistically significant. The PAF for low HDL-C was the highest in men (63.2%) and in rs3764261 (31.5%) of the genetic factors, and the PAFs of smoking and drinking were 23.1% and 41.8%, respectively.

**Conclusion:**

The present study showed that the population-based impact of genomic factor *CETP* rs3764261 for low HDL-C was higher than that of smoking and lower than that of drinking.

## INTRODUCTION

Low serum levels of HDL-cholesterol (HDL-C) are associated with an increased risk of cardiovascular disease (CVD).^1^^,^^2^ As clinically available drugs that can enhance HDL-C levels are limited, genetic and environmental factors play an important role in the alleviation of CVD risk. Smoking, alcohol intake, physical activity, BMI, and diet intake have been confirmed to be environmental factors that affect HDL-C levels.^3^^–^^6^

The effects of genetic factors, such as single nucleotide polymorphism (SNPs) in various enzymes-encoding genes, on HDL-C levels have also been reported.^7^ Although the regulation of HDL-C metabolism is a complex process, enzymes in the reverse cholesterol transport (RCT) system, such as ABCA1, LCAT, cholesteryl ester transfer protein (CETP), hepatic lipase (LIPC), APOA1/C3/A4/A5, scavenger receptor class B type I (SCARB1), and LPL, play a major role in it.^2^ Multiple SNPs have been reported to be associated with HDL-C levels, and among the genes harboring such SNPs, the genetic variants of *CETP* have been observed to exert a greater influence on HDL-C levels.^8^^–^^11^ Furthermore, besides association with SNPs in RCT-related genes, the association with several other SNPs, such as those in genes encoding endothelial lipase (LIPG) and APOE, which are related to lipoprotein dynamism, has been reported.^10^^,^^12^

The majority of studies on environmental and genetic factors that affect HDL-C levels focus on factor-based association with respect to individual risk and susceptibility, and the population-based impact of environmental and genetic factors on HDL-C levels has not been clearly evaluated. The population-based impact of a factor is an important aspect for public health. The population-based impact of various environmental factors on HDL-C levels can be estimated based on the impact of the association and prevalence of each factor. However, the population-based impact of genetic factors is difficult to estimate, because several SNPs are detected in each enzyme-encoding gene; the impact of the association of each SNP with HDL-C levels will differ, and the prevalence of the allele containing each SNPs will differ as well. Therefore, studies that investigate the combined effect of HDL-C-related SNPs limit their assessment to certain representative SNPs.^9^ Furthermore, gene-environment interaction may influence HDL-C levels as well.^13^^,^^14^

Among environmental factors, smoking and drinking habits significantly affect the reduction or increase in HDL-C levels, respectively.^2^^,^^9^^,^^15^ These factors are suitable candidates for the estimation of the population-based impact of environmental factors on HDL-C levels, while also taking into account the interaction with genetic factors. In such cases, GWAS are suitable for evaluating the overall scenario. GWAS on the effects of HDL-C-related SNPs on ethnic populations, including the Japanese population, have been performed earlier, and all HDL-C-related SNPs have been listed in the catalog.^16^^,^^17^

To investigate the population-based impact of smoking, drinking, and genetic factors on low HDL-C, we conducted a relatively large-sized cross-sectional study using data on environmental factors and GWAS from the Japan Multi-Institutional Collaborative Cohort (J-MICC) Study.

## METHODS

### Study population

The J-MICC Study was a large-scaled cohort study that commenced in 2005; it investigated the host- and environment-related factors that affect cancer and other lifestyle-related diseases.^18^^–^^20^ In brief, data on the lifestyles and medical history of patients were collected using questionnaires, while blood samples and health checkup results were collected during the baseline survey after written informed consent was obtained. The participants were recruited from among health-checkup examinees by the local government, private companies, and health checkup centers; responders who posted responses to regional residents and first-visit outpatients at cancer center. The subjects (*n* = 14,555) of the GWAS selected from among the J-MICC Study participants were aged from 35–69 years and belonged to 11 prefectures of Japan (Chiba, Shizuoka, Aichi, Shiga, Kyoto, Tokushima, Fukuoka, Saga, Nagasaki, Kagoshima, and Okinawa); participants were selected by ten research institutes and universities. The present study excluded data that did not include information on HDL-C levels (all participants [*n* = 2,296] from the Chiba study region and the Aichi Cancer Center and some participants [*n* = 187] from other institutes), smoking (*n* = 180), and drinking (*n* = 24); and from cases of withdrawal (*n* = 21). Data from certain subjects qualified for multiple exclusion criteria. The final number of eligible subjects was 11,498 (the dataset used in the present study was decided upon on March 12, 2020, version 20200312).

The ethics committees of Nagoya University Graduate School of Medicine, Kagoshima University Graduate School of Medical and Dental Sciences, and other participating institutes and universities approved the protocol.

### Questionnaire survey

A standardized structured questionnaire was used in the J-MICC Study to collect information regarding lifestyle factors and medical history of the subjects.^19^ The questionnaire was evaluated by trained staff to ensure completeness and consistency.

### HDL-C level assessment

Venous blood samples were collected from the subjects in sitting position during a period of fasting. The mean duration of fasting was 9.8 h. The blood samples were separated into serum, plasma, and buffy coat fractions, and stored directly at −80°C on the day of sampling. The serum HDL-C levels were measured at the respective institutes for health checkup or medical examination in each study region.^21^

### Quality of samples and SNPs during genotyping

DNA was extracted from the buffy coat fractions using a BioRobot M48 Workstation (Qiagen Group, Tokyo, Japan) at Nagoya University, using samples from all regions except Fukuoka and KOPS (Kyushu and Okinawa Population Study); DNA was extracted from the samples from these two regions at Kyushu University using an automatic nucleic acid isolation system (NA-3000; Kurabo, Co., Ltd, Osaka, Japan). Next, the DNA samples were genotyped at the RIKEN Center for Integrative Medicine using a HumanOmniExpressExome-8 v1.2 BeadChip array (Illumina Inc., San Diego, CA, USA). The number of low-quality DNA samples was 463, which were excluded from the analysis. The subjects for whom sex information in the questionnaire was inconsistent with that revealed by the genotyping results were excluded. Furthermore, the identity-by-descent method implemented in the PLINK 1.9 software^22^ was used to identify close relationship pairs (pi-hat >0.1875) and the sample from each pair was excluded. The subjects (*n* = 34) with non-Japanese estimated ancestries^23^ were also excluded by principal component analysis (PCA)^24^ using a 1,000 Genomes reference panel (phase 3).^25^

SNPs with a genotype call rate <0.98, a Hardy-Weinberg equilibrium exact test *P*-value <1 × 10^−6^, and a low minor allele frequency (MAF) <0.01, or a departure from the allele frequency computed from the 1,000 Genomes Phase 3 EAS (East Asian) samples; and non-autosomal SNPs were excluded. Such quality control filtering resulted in 14,091 individuals and 570,162 SNPs.

### Genotype imputation and post-imputation quality control

The imputation of genotypes in autosomal chromosomes was performed using SHAPEIT2^26^ and Minimac3^27^ software with the 1,000 Genomes reference panel (phase 3).^25^ The imputation procedure displayed 47,109,431 SNPs from 570,162 SNPs.

The SNPs with imputation quality *r*^2^ < 0.3 were excluded in the post-imputation quality control step. The number of eligible SNPs was 12,617,547.

### Selection of HDL-C-related SNPs

**Figure 1.**
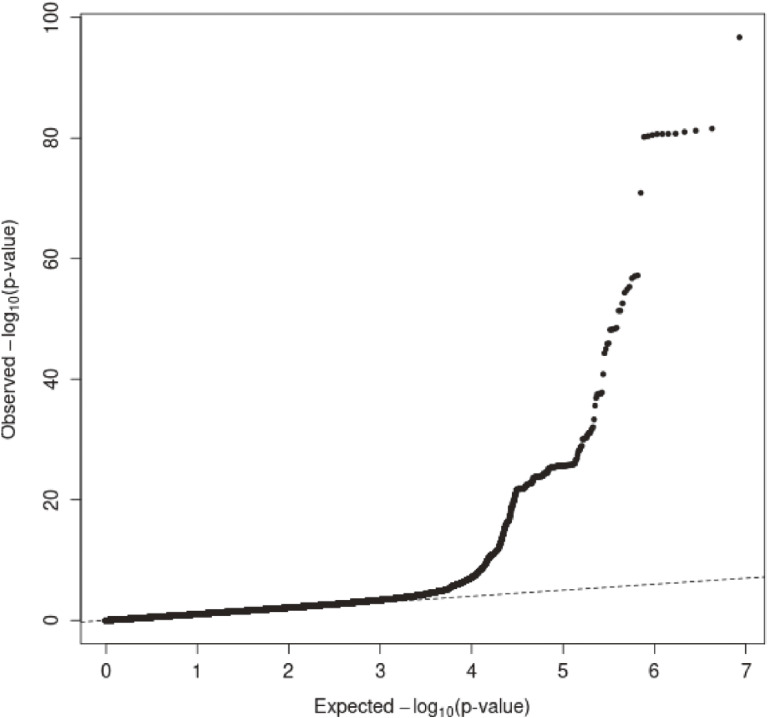
Q-Q plot for *P* values from original GWAS data. The vertical and horizontal axes indicate observed and expected −log_10_ (*P* value) for tests of association between SNPs and HDL-C, respectively. GWAS, genome-wide association study; HDL-C, high-density lipoprotein cholesterol; SNP, single-nucleotide polymorphism.

On August 27, 2019, HDL-C-related SNPs were systematically selected from the GWAS catalog (https://www.ebi.ac.uk/gwas/) (the database of published GWAS), which included 499 SNPs from all ethnic population.^16^^,^^17^ Next, 65 SNPs among these were selected for the present study, which had *P*-values of genome-wide significance (*P* < 5 × 10^−8^) in the present analysis (eTable 1). The Q-Q plot showed the apparently different distribution of the present observed log_10_ (*P*-value) of the 65 SNPs against the expected log_10_ (*P*-value) (Figure 1). Although the association for rs921919 in *SCARB1* (12q24.31) indicated genome-wide significance, this was not included in the present analysis because this SNP was not previously reported to be associated with HDL-C levels and were not listed in the GWAS catalog. Other SNPs in *SCARB1* listed in the GWAS catalog were not genome-wide significant in the present analysis.

### Statistical analysis

The subjects were divided into two categories based on the smoking status (“never” and “former” [≥1 year] vs “current” [include smokers within 1 year after quitting]), because the HDL-C levels apparently differed between subjects with the “current” and “never” statuses, and with respect to the duration after quitting. The subjects were also divided into two categories based on the drinking status (non-, former, and current moderate drinkers [<20 g/day] vs current heavy drinkers [≥20 g/day]), as the Japanese Ministry of Health, Labour and Welfare recommends alcohol intake in moderation (at <20 g/day); the HDL-C levels apparently differed between the two categories.^28^ The duration and intensity of daily activity (hard work and walking) and the frequency and intensity of habitual exercise were used to estimate the metabolic equivalents (METs). The estimation of METs·hour per day was based on the duration and intensity of exercise, with 3.0 for walking, 3.3 for light exercise, 4.0 for moderate exercise, 4.5 for heavy work, and 8.0 for heavy exercise.^29^ Daily activity was classified as <8.25 METs·h/day and ≥8.25 METs·h/day at the median value. Habitual exercise was classified as <0.728 METs·h/day and ≥0.728 METs·h/day at the median value. Egg intake was selected as a representative HDL-C-related dietary factor.^2^^,^^9^ There were two categories for BMI with comparable number of male and female subjects in each. The association between HDL-C levels (continuous) and non-genetic factors, such as smoking and drinking habits, was tested using multivariate linear regression analysis after adjusting for the following HDL-C-related factors: age (<57 vs ≥57 years), sex, smoking, drinking, daily activity, habitual exercise, egg intake, and BMI. Dummy variables of 0 and 1 were used for all independent variables. Statistical analyses for non-genetic factors were performed using Stata software (version 12; Stata Corp., College Station, TX, USA), and differences with *P*-value <0.05 were considered statistically significant.

**Figure 2.**
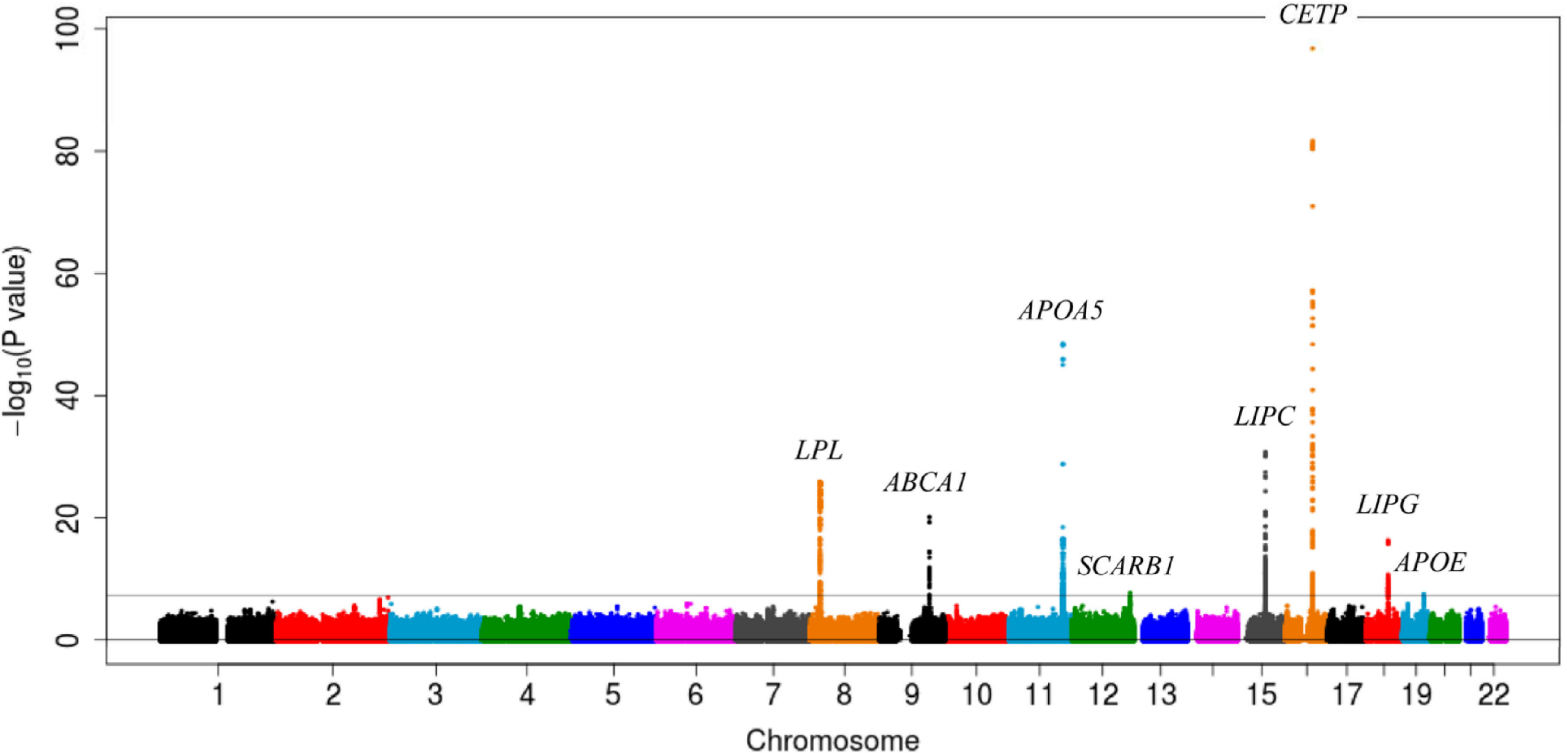
Manhattan plot (−log_10_ of the *P* value based on genomic location) of the association between the SNPs denoted in the original GWAS and the HDL-C levels shows the formation of eight peaks over the line representing *P* < 5 × 10^−8^ for *LPL* (8p21.3), *ABCA1* (9q31.1), *APOA5* (11q23.3), *SCARB1* (12q24.31), *LIPC* (15q21.3), *CETP* (16q13), *LIPG* (18q21.1), and *APOE* (19q13.32). The horizontal line represents the genome-wide significance level (α = 5 × 10^−8^). GWAS, genome-wide association study; HDL-C, high-density lipoprotein cholesterol; SNP, single-nucleotide polymorphism.

The selected HDL-C-related 65 SNPs were divided into seven categories based on the gene and cytoBand groups (eTable 1). The Manhattan plot for total SNPs in the present GWAS consistently showed seven peaks with genome-wide significance, with the exception of a single peak corresponding to rs921919 in *SCARB1* with genome-wide significance yet unlisted in the GWAS catalog (Figure 2). Next, the seven SNPs with the highest coefficients and lowest *P*-values from each of the seven groups were selected. The association between HDL-C levels (continuous) and genetic factors, and the interaction were tested using multivariate linear regression analysis in epacts v3.2.6 software (https://genome.sph.umich.edu/wiki/EPACTS), after adjusting for the HDL-C-related factors and first five principal components. Dummy variables of 0, 0.5, and 1 were used for the number of alternative alleles (0, 1, and 2) as independent variables in order to compare the impact of coefficients on non-genetic factors (dummy variables of 0 and 1), and the coefficients and 95% confidence intervals (CIs) were estimated. Differences with α = 5 × 10^−8^ were considered statistically significant in the GWAS. We applied the Bonferroni correction (*P* < 0.00077) for evaluating the differences in interaction of smoking or drinking with the 65 SNPs to reduce the chances of introducing an alpha error by multiple hypothesis testing.

The population-based impact of the non-genetic and genetic factors was estimated using population attributable fraction (PAF).^30^^,^^31^ First, the odds ratio (OR) for low HDL-C (<40 mg/dL) was estimated, and the PAF was calculated as;

$$PAF=P\times \frac{\left(OR-1\right)}{OR}\times 100\;(\%)$$

where *P* is the proportion of the exposure in subjects with low HDL-C. The reference exposure group was defined as those with the minimum risk for low HDL-C, ie smoking habit (“never” and “former” [≥1 year]), drinking habit (≥20 gram alcohol/day), daily activity (≥8.25 METs/day), habitual exercise (≥0.73 METs/day), egg intake (≥3 times/week), BMI (<23.0 kg/m^2^), age (<57 years), and sex (women) in the non-genetic factors; and rs3764261, rs662799, rs1800588, rs328, and rs3786247 (referent and alterative allele hetero-genotype, and alterative allele homo-genotype), as well as rs2575876 and rs429358 (referent allele homo-genotype), in the genetic factors. Dummy variables of 0 and 1 were used for both the non-genetic and genetic factors. When the PAF of the combined SNPs was estimated, the accumulation in 6 SNPs was categorized according to the number of the high-risk genotypes for low HDL-C by individual regardless kind of SNPs (ie, 0–1 SNPs for reference, 2 SNPs, 3 SNPs and 4–6 SNPs). The SNP of rs1800588 was excluded from this accumulation analysis, because the OR for low HDL-C was not statistically significant. The ORs and their 95% CIs were estimated using logistic model after adjusting for age, sex, smoking, drinking, daily activity, habitual exercise, egg intake, and BMI.

## RESULTS

The distribution pattern of male and female subjects in the two age groups (35–56 years and 57–69 years) was almost similar (Table 1). The prevalence of current smokers was 34.9% among male and 7.3% among female subjects (19.7% in both), and that of heavy drinkers was 42.7% in males and 5.1% in females (22.0% in both). The prevalence of low HDL-C (<40 mg/dL) was 8.9% in male and 1.8% in female subjects (5.0% in both sexes).

**Table 1.** Age-, environmental factor-, BMI-, and HDL-C level-based distribution of study subjects divided by sex

	Number (%)			

	Men		Women	
Age, years				
35–56	2,595	(50.7)	3,280	(52.4)
57–69	2,519	(49.3)	2,976	(47.6)
Total	5,114	(100)	6,256	(100)
Smoking				
Never and former (≥1 year) smokers	3,329	(65.1)	5,802	(92.7)
Current smokers^a^	1,785	(34.9)	454	(7.3)
Drinking				
Non-, former and moderate drinkers^b^	2,933	(57.4)	5,936	(94.9)
Heavy drinkers^c^	2,181	(42.7)	320	(5.1)
Daily activity				
<8.25 METs·h/day	3,102	(60.7)	3,484	(55.7)
≥8.25 METs·h/day	2,012	(39.3)	2,772	(44.3)
Habitual exercise				
<0.73 METs·h/day	2,447	(47.9)	3,268	(52.2)
≥0.73 METs·h/day	2,667	(52.2)	2,988	(47.8)
Egg intake				
<3 times/week	3,659	(71.6)	4,377	(70.0)
≥3 times/week	1,455	(28.5)	1,879	(30.0)
BMI, kg/m^2^				
<23	2,066	(40.4)	3,789	(60.6)
≥23	3,048	(59.6)	2,467	(39.4)
HDL-C				
<40 mg/dL	454	(8.9)	111	(1.8)
≥40 mg/dL	4,660	(91.1)	6,145	(98.2)

BMI, body mass index; HDL-C, high-density lipoprotein cholesterol; METs, metabolic equivalents.

^a^Smokers within 1 year after quitting were included.

^b^<20 g alcohol/day.

^c^≥20 g alcohol/day.

Drinking (*P* < 0.001), daily activity (*P* < 0.001), habitual exercise (*P* < 0.001), egg intake (*P* = 0.004), and sex (*P* < 0.001) were associated positively with the HDL-C levels, while smoking (*P* < 0.001), BMI (*P* < 0.001), and age (*P* < 0.001) were associated negatively (Table 2).

**Table 2.** Association between HDL-C levels and environmental factors determined in multivariate regression analysis

	Coeff.^a^	95% CI	*P*-value
Smoking (current)	−5.407	−6.133 to −4.681	*<0.001*
Drinking (≥20 g alcohol/day)	7.274	6.549 to 8.000	*<0.001*
Daily activity (≥8.25 METs·h/day)	1.033	0.493 to 1.574	*<0.001*
Habitual exercise (≥0.73 METs·h/day)	1.480	0.938 to 2.021	*<0.001*
Egg intake (≥3 times/week)	0.856	0.270 to 1.442	*0.004*
BMI (≥23.0 kg/m^2^)	−8.738	−9.283 to −8.194	*<0.001*
Age (≥57 years)	−1.496	−2.040 to −0.952	*<0.001*
Sex (women)	9.567	8.931 to 10.203	*<0.001*

BMI, body mass index; CI, confidence interval; Coeff., coefficient; HDL-C, high density lipoprotein cholesterol; METs, metabolic equivalents.

^a^Adjusted for age, sex, smoking, drinking, daily activity, habitual exercise, egg intake, and BMI. The coefficient value represents change in HDL-C per dummy variable (0, 1) of environmental factors.

The seven major SNPs selected from the 65 SNPs in the GWAS catalog according to the gene and cytoBand groups were rs3764261 in *HERPUD1–CETP* (16q13), rs662799 in *APOA5* (11q23.3), rs1800588 in *LIPC* (15q21.3), rs328 in *LPL* (8p21.3), rs2575876 in *ABCA1* (9q31.1), rs3786247 in *LIPG* (18q21.1), and rs429358 in *APOE* (19q13.32) (Table 3). The frequencies (0.100 to 0.649) and coefficients (−4.003 to 8.863) varied for each SNP, and the highest coefficient was observed for rs3764261.

**Table 3.** Multivariate regression analysis between HDL-C levels and seven HDL-C related SNPs from the GWAS catalog

SNP	cytoBand	REF/ALT	Gene	Frequency of ALT	Coeff.^a^	95% CI	*P*-value
rs3764261	16q13	C/A	*HERPUD1*, *CETP*	0.207	8.863	7.958 to 9.770	6.07 × 10^−82^
rs662799	11q23.3	G/A	*APOA5*	0.649	5.713	4.932 to 6.494	1.12 × 10^−46^
rs1800588	15q21.3	C/T	*LIPC*	0.510	4.447	3.700 to 5.194	1.76 × 10^−31^
rs328	8p21.3	C/G	*LPL*	0.126	6.136	5.006 to 7.266	1.77 × 10^−26^
rs2575876	9q31.1	G/A	*ABCA1*	0.276	−4.003	−4.840 to −3.164	7.67 × 10^−21^
rs3786247	18q21.1	T/G	*LIPG*	0.460	3.209	2.452 to 3.966	1.02 × 10^−16^
rs429358	19q13.32	T/C	*APOE*	0.100	−3.594	−4.864 to −2.322	2.89 × 10^−8^

ALT, alternative allele; BMI, body mass index; CI, confidence interval; Coeff., coefficient; GWAS, genome-wide association study; HDL-C, high density lipoprotein cholesterol; REF, referent allele; SNP, single nucleotide polymorphism.

^a^Adjusted for age, sex, smoking, drinking, daily activity, habitual exercise, egg intake and BMI. The coefficient value represents change in HDL-C per ALT allele copy (0, 1, 2) for the SNP.

The HDL-C levels varied for each genotype group based on the smoking and drinking status (Table 4). The highest HDL-C level (mean 74.6; 95% CI, 70.8–78.4 mg/dL) was observed in heavy drinkers with the rs3764261 alternative homo-genotype (AA), while the lowest was observed in current smokers with the rs662799 referent homo-genotype (GG) and hetero-genotype (GA). The gene-environment interactions between the seven SNPs and smoking/drinking were not statistically significant, and the lowest *P*-value of 0.004 was higher than the *P*-value obtained after applying Bonferroni correction (*P* < 0.00077). These interactions were not statistically significant for all 65 SNPs selected from the GWAS catalog (eTable 1). No significant interaction was observed in the subgroup analysis based on sex (data not shown in eTable 1).

**Table 4.** Interaction between HDL-C levels according to different smoking and drinking statues and the 7 selected SNPs

	Smoking					Drinking				
	
	Never & former		Current		*P*-value for interaction^a^	Non-moderate		Heavy^b^		*P*-value for interaction^a^
			
	RR & RA	AA	RR & RA	AA		RR & RA	AA	RR & RA	AA	
	
	HDL-C (mg/dL)	HDL-C (mg/dL)	HDL-C (mg/dL)	HDL-C (mg/dL)		HDL-C (mg/dL)	HDL-C (mg/dL)	HDL-C (mg/dL)	HDL-C (mg/dL)	
rs3764261	64.0	72.2	56.0	64.7	0.156	62.5	69.5	62.2	74.6	0.015
	(63.7–64.3)	(70.6–73.8)	(55.4–56.6)	(60.7–68.8)		(62.1–62.8)	(67.9–71.2)	(61.6–62.9)	(70.8–78.4)	
	*N* = 8,723	*N* = 408	*N* = 2,130	*N* = 109		*N* = 8,464	*N* = 405	*N* = 2,389	*N* = 112	
rs662799	62.9	66.5	54.7	58.5	0.706	61.5	64.6	60.7	65.6	0.004
	(62.4–63.3)	(66.0–67.0)	(53.9–55.5)	(57.5–59.5)		(61.0–61.9)	(64.1–65.1)	(59.9–61.6)	(64.5–66.6)	
	*N* = 5,301	*N* = 3,830	*N* = 1,236	*N* = 1,003		*N* = 5,094	*N* = 3,775	*N* = 1,443	*N* = 1,058	
rs1800588	63.6	66.6	55.3	59.5	0.312	62.0	65.0	61.7	65.8	0.387
	(63.2–64.0)	(65.9–67.2)	(54.6–56.0)	(58.2–60.8)		(61.6–62.4)	(64.4–65.7)	(61.0–62.5)	(64.5–67.2)	
	*N* = 6,723	*N* = 2,408	*N* = 1,661	*N* = 578		*N* = 6,530	*N* = 2,339	*N* = 1,854	*N* = 647	
rs328	64.3	70.4	56.4	58.4	0.735	62.7	69.0	62.8	64.8	0.658
	(63.9–64.6)	(67.6–73.2)	(55.7–57.0)	(52.3–64.4)		(62.4–63.0)	(66.1–72.0)	(62.1–63.4)	(59.6–70.1)	
	*N* = 8,983	*N* = 148	*N* = 2,207	*N* = 32		*N* = 8,721	*N* = 148	*N* = 2,469	*N* = 32	
rs2575876	64.6	62.1	56.6	54.6	0.476	63.0	60.5	63.0	61.0	0.354
	(64.2–64.9)	(61.0–63.3)	(55.9–57.2)	(52.4–56.7)		(62.6–63.3)	(59.3–61.6)	(62.3–63.6)	(58.6–63.4)	
	*N* = 8,436	*N* = 695	*N* = 2,062	*N* = 177		*N* = 8,207	*N* = 662	*N* = 2,291	*N* = 210	
rs3786247	63.9	66.0	55.6	59.3	0.670	62.3	64.6	62.2	65.0	0.569
	(63.6–64.3)	(65.3–66.7)	(55.0–56.3)	(57.8–60.7)		(62.0–62.7)	(63.8–65.3)	(61.5–62.9)	(63.5–66.5)	
	*N* = 7,232	*N* = 1,899	*N* = 1,770	*N* = 469		*N* = 7,029	*N* = 1,840	*N* = 1,973	*N* = 528	
rs429358	64.4	61.6	56.4	57.9	0.931	62.8	60.7	62.8	61.1	0.723
	(64.1–64.7)	(58.1–65.0)	(55.8–57.0)	(50.1–65.6)		(62.5–63.2)	(57.4–64.0)	(62.1–63.5)	(52.3–70.0)	
	*N* = 9,037	*N* = 94	*N* = 2,214	*N* = 25		*N* = 8,773	*N* = 96	*N* = 2,478	*N* = 23	

AA, alterative homo-genotype; BMI, body mass index; HDL-C, high density lipoprotein cholesterol; RA, referent and alterative allele hetero-genotype; RR, referent allele homo-genotype; SNP, single nucleotide polymorphism.

^a^Adjusted for age, sex, smoking, drinking, daily activity, habitual exercise, egg intake, and BMI.

^b^≥20 g alcohol/day.

The ORs for low HDL-C were statistically significant for several non-genetic factors, including smoking, drinking, BMI, age and sex, and for the genetic factors, and six of the seven SNPs (except rs1800588) (Table 5). The PAF for low HDL-C in the non-genetic factors was the highest in men (63.2%), and the PAFs of smoking and drinking were 23.1% and 41.8%, respectively. The PAF for low HDL-C in the genetic factors was the highest in rs3764261 (31.5%), which was higher than that of smoking and lower than that of drinking. The impact of the PAFs of three SNPs (25.5%) and 4–6 SNPs (23.7%) according to the number of SNPs with high-risk genotype for low HDL-C was similar to that of smoking, although the ORs for low HDL-C showed an apparent increasing trend with the number of SNPs with higher-risk genotype (*P* < 0.001).

**Table 5.** Population attributable fractions of non-genetic and genetic factors for low HDL-C

	Proportion of exposure in low HDL-C subjects (%)	OR^a^	95% CI	PAF (%)
Non-genetic factors				
Smoking habit (current)	41.8	2.23	1.85–2.70	23.1
Drinking habit (<20 grams alcohol/day)	76.8	2.19	1.77–2.71	41.8
Daily activity (<8.25 METs/day)	62.7	1.11	0.93–1.33	—
Habitual exercise (<0.73 METs/day)	52.4	1.14	0.95–1.36	—
Egg intake (<3 times/week)	70.4	0.93	0.77–1.12	—
BMI (≥23.0 kg/m^2^)	72.6	2.35	1.93–2.85	41.6
Age (≥57 years)	54.5	1.44	1.20–1.72	16.6
Sex (men)	80.4	4.68	3.72–5.89	63.2
Genetic factors				
rs3764261 (RR)	73.5	1.75	1.44–2.13	31.5
rs662799 (RR)	26.6	2.89	2.35–3.55	17.4
rs1800588 (RR)	27.3	1.16	0.95–1.41	—
rs328 (RR)	81.1	1.36	1.09–1.70	21.6
rs2575876 (RA & AA)	55.6	1.43	1.20–1.71	16.8
rs3786247 (RR)	34.5	1.36	1.13–1.64	9.2
rs429358 (RA & AA)	24.6	1.56	1.27–1.92	8.9
Number of SNPs with high-risk genotype^b^				
0–1 SNPs	7.3	1.00	—	—
2 SNPs	26.4	1.97	1.38–2.82	13.0
3 SNPs	37.4	3.16	2.24–4.47	25.5
4–6 SNPs	29.0	5.49	3.84–7.84	23.7
*P* for trend		*<0.001*		

AA, alterative allele homo-genotype; BMI, body mass index; CI, confidence interval; HDL-C, high density lipoprotein cholesterol; OR, odds ratio; PAF, population attributable fraction; RA, referent and alterative allele hetero-genotype; RR, referent allele homo-genotype; SNPs, single nucleotide polymorphisms.

^a^Adjusted for age, sex, smoking, drinking, daily activity, habitual exercise, egg intake and BMI.

^b^SNP of rs1800588 is excluded.

## DISCUSSION

In the present study, we observed significant associations between HDL-C levels and smoking, drinking, daily activity, habitual exercise, egg intake, BMI, age, sex, and seven SNPs in *CETP*, *APOA5*, *LIPC*, *LPL*, *ABCA1*, *LIPG*, and *APOE*. The PAFs, as a population-based impact, for low HDL-C were the highest in men on the non-genetic factors and in *CETP* rs3764261 on the genetic factors. The impact of the genetic factor PAF was higher than that of smoking and was lower than that of drinking.

Genetic factors that affect HDL-C levels, such as SNPs, are primarily associated with genes that encode enzymes from the RCT system, such as *ABCA1*, *LCAT*, *CETP*, *LIPC*, *APOA1/C3/A4/A5*, *SCARB1*, and *LPL*.^2^^,^^7^ The SNPs in the corresponding genes, except those in *LCAT* and *SCARB1*, were considered among the seven major SNPs selected in the present analysis. The SNPs in *SCARB1* were not included because the two SNPs with genome-wide significance were not listed in the GWAS catalog, and the lowest *P*-value for the *SCARB1* SNP (rs838886) listed in the catalog was higher than the genome-wide significance (*P* = 7.34 × 10^−8^; data not shown in eTable 1). As the MAF of *LCAT* was less than 0.01, the SNPs of *LCAT* were excluded from the GWAS analysis. The SNPs in *LIPG* and *APOE*, which are associated with HDL-C production via a system different from RCT, were also considered among the seven major SNPs.^10^^,^^12^ The genetic variants of *CETP* were observed to exhibit the most significant influence on HDL-C levels, which was concordant with findings from previous reports.^8^^–^^10^

Cigarette smoking is associated with lower HDL-C levels, even though the mechanisms are yet to be completely elucidated. Certain studies have shown that smoking is related to ApoA1 concentration^13^ and CETP activity^14^; however, these results could be considered controversial.^32^^,^^33^ Alcohol consumption is reported to be associated with increased expression of ABCA1^34^ and a higher APOA1 concentration^35^ in peripheral blood and a lower CETP activity.^36^

In the present study, the interaction of the 65 and seven SNPs with drinking was not statistically significant after Bonferroni correction was applied. Previous studies reported significant association of alcohol consumption and polymorphisms in multiple genes (*CETP*, *APOA1*/*A2*, *LPL*, *ADH3*, *ADH1*, and *ALDH2*) with HDL-C levels.^37^^–^^41^ The association between CETP and ABCA1 expressions, and alcohol consumption has been also reported in previous studies, but their mechanism is not clear.^34^^,^^36^ However, no genome-wide significance was reported in the gene-alcohol interaction for *CETP*, *APOA5*, *LIPC*, and *LPL* in a particular GWAS.^42^ The interaction between each SNP and smoking was also not statistically significant after Bonferroni correction was applied. These results suggest that genetic factors may have a minor or negligible impact on the interaction with drinking and smoking.

Several studies have previously reported the association between SNPs and HDL-C levels, which have been listed in the GWAS catalog. In the present study, we selected the 498 SNPs listed in the GWAS results that were a part of the J-MICC Study and observed 65 SNPs with genome-wide significance for the analysis. We selected seven SNPs according to the gene and cytoBand groups. The Manhattan plot for total SNPs consistently showed seven peaks, except that for *SCARB1*. These observations support proposition that the seven SNPs are appropriate representatives of the SNPs associated with HDL-C levels in the present analysis.

In the present study, we investigated the population-based impact of both non-genetic and genetic factors on low HDL-C, using PAF. The OR for low HDL-C was used as the relative risk when the PAF was calculated, because the prevalence of low HDL-C was obtained from the baseline general population and its rate was relatively low (5.0% in both sexes).^30^^,^^31^ To the best of our knowledge, studies investigating the PAF for low HDL-C with non-genetic and/or genomic factors have not yet been conducted. The highest PAFs was observed in men on the non-genetic factors and in *CETP* rs3764261 on the genetic factors. The impact of the genetic factor PAF was higher than that of smoking and was lower than that of drinking. These observations suggest that, from a public health perspective, the population-based impact of genomic factors for low HDL-C is comparably high compared to non-genetic factors.

The strength of this study is that the population-based impact of non-genetic and genetic factors on HDL-C levels was evaluated simultaneously using data from an adequate number of subjects and total gene information. To our knowledge, this is the first comprehensive report on the population-based impact of the abovementioned factors.

Meanwhile, the present study has several limitations. First, a causal relationship was not confirmed, as this is a cross-sectional study. Second, atheroprotective and non-atheroprotective HDL particles were jointly considered as total HDL-C. The two fractions of HDL2-C and HDL3-C have different effects on CVD risk.^2^ Third, the present study selected seven representative SNPs to estimate the population-based impact; the highest impact may have been estimated because the highest coefficients of the seven representative SNPs were selected based on the gene and cytoBand groups. Fourth, the replication test on GWAS was not conducted, because the present study used information from the GWAS catalog in which the association between SNPs and HDL-C levels had been estimated and published previously. Fifth, the effect of residual SNPs (those apart from the 65 SNPs), referred to as “missing heritability”, was not considered. The polygenic risk score may support the estimation of this effect.^43^ Sixth, PAF valid only in the absence of confounding and/or effect modification.^30^ The lack of unknown data on confounding is likely to misestimate the true PAF, the extent to which is dependent on the magnitude of confounding.^31^ Furthermore, PAF estimate is restricted by time and population and depends on the quality and representativeness of the exposure and risk data.

In conclusion, the present study demonstrated that the population-based impact of genomic factor *CETP* rs3764261 for low HDL-C was higher than that of smoking and lower than that of drinking.

## SUPPLEMENTARY MATERIAL

eTable 1. Sixty-five HDL-C related SNPs selected from the GWAS catalog, and their association with HDL-C levels and interaction with smoking and drinking
