# Supplementary material for: Population-Based Impact of Smoking, Drinking, and Genetic Factors on HDL-cholesterol Levels in J-MICC Study Participants
Source: J Epidemiol. 2023 Apr 5;33(4):193–200. doi: 10.2188/jea.JE20210142 (PMC9939920; doi:10.2188/jea.JE20210142)
Supplement: Supplementary file 1 [file je-33-193-s001.pdf]

eTable 1. Sixty-five HDL-C related SNPs selected from the GWAS catalog, and their association with HDL-C levels and interaction with smoking and drinking

SNPs	cytoBand	REF/ ALT	Gene	Frequency of ALT	Association with HDL-C levels		Interaction with smoking			Interaction with drinking		
					Coeff.[a]	*P*-value	Coeff.[a]	SE	*P*-value	Coeff.[a]	SE	*P*-value
rs328	8p21.3	C/G	*LPL*	0.126	6.136	1.77×10^{-26}	-0.247	0.730	0.735	-0.310	0.701	0.658
rs325	8p21.3	T/C	*LPL*	0.126	6.131	2.03×10^{-26}	-0.249	0.731	0.734	-0.296	0.701	0.673
rs79407615	8p21.3	T/G	*LPL, SLC18A1*	0.125	6.141	2.18×10^{-26}	-0.135	0.733	0.854	-0.133	0.704	0.85
rs12678919	8p21.3	A/G	*LPL, SLC18A1*	0.125	6.131	2.21×10^{-26}	-0.081	0.732	0.912	-0.168	0.704	0.811
rs10503669	8p21.3	C/A	*LPL, SLC18A1*	0.125	6.129	2.69×10^{-26}	-0.128	0.733	0.861	-0.127	0.704	0.857
rs7841189	8p21.3	C/T	*LPL, SLC18A1*	0.125	6.105	4.27×10^{-26}	-0.113	0.733	0.877	-0.186	0.704	0.792
rs10096633	8p21.3	C/T	*LPL, SLC18A1*	0.125	6.084	6.24×10^{-26}	-0.126	0.733	0.863	-0.201	0.704	0.775
rs17482753	8p21.3	G/T	*LPL, SLC18A1*	0.125	6.071	7.96×10^{-26}	-0.118	0.733	0.872	-0.193	0.704	0.784
rs13702	8p21.3	T/C	*LPL*	0.186	5.087	3.23×10^{-25}	0.628	0.624	0.314	0.209	0.596	0.726
rs15285	8p21.3	C/T	*LPL*	0.186	5.087	3.23×10^{-25}	0.628	0.624	0.314	0.209	0.596	0.726
rs287	8p21.3	A/G	*LPL*	0.192	5.141	4.56×10^{-25}	0.434	0.632	0.493	0.062	0.604	0.918
rs2083637	8p21.3	A/G	*LPL, SLC18A1*	0.186	5.021	1.19×10^{-24}	0.630	0.623	0.312	0.199	0.597	0.739
rs326	8p21.3	A/G	*LPL*	0.188	4.981	2.15×10^{-24}	0.576	0.621	0.354	0.196	0.594	0.741
rs11984636	8p21.3	T/C	*LPL, SLC18A1*	0.118	5.761	2.01×10^{-22}	0.195	0.748	0.794	-0.174	0.714	0.808
rs115849089	8p21.3	G/A	*LPL, SLC18A1*	0.130	5.313	1.35×10^{-19}	0.409	0.750	0.586	0.111	0.714	0.877
rs9644568	8p21.3	G/A	*LPL, SLC18A1*	0.128	5.673	1.16×10^{-18}	0.356	0.827	0.667	0.092	0.780	0.906
rs4244457	8p21.3	C/T	*LPL, SLC18A1*	0.268	3.305	1.84×10^{-14}	0.761	0.557	0.172	0.619	0.526	0.239

rs28526159	8p21.3	C/T	*LPL, SLC18A1*	0.274	3.075	7.75×10^{-13}	0.760	0.553	0.169	0.439	0.523	0.401
rs2575876	9q31.1	G/A	*ABCA1*	0.276	-4.003	7.67×10^{-21}	0.381	0.535	0.476	0.472	0.509	0.354
rs1883025	9q31.1	C/T	*ABCA1*	0.283	-3.97	8.59×10^{-21}	0.384	0.531	0.470	0.648	0.506	0.201
rs2740488	9q31.1	A/C	*ABCA1*	0.279	-3.904	5.62×10^{-20}	0.386	0.534	0.470	0.376	0.509	0.46
rs12686004	9q31.1	G/A	*ABCA1*	0.234	-3.409	3.16×10^{-14}	0.247	0.564	0.661	0.561	0.534	0.293
rs4149268	9q31.1	C/T	*ABCA1*	0.357	-2.713	1.20×10^{-11}	0.168	0.507	0.741	0.190	0.476	0.689
rs662799	11q23.3	G/A	*APOA5*	0.649	5.713	1.12×10^{-46}	0.188	0.498	0.706	1.377	0.480	0.004
rs651821	11q23.3	C/T	*APOA5*	0.648	5.684	1.34×10^{-46}	0.187	0.496	0.706	1.350	0.478	0.005
rs11216126	11q23.3	A/C	*LOC101929011, BUD13*	0.157	4.271	1.93×10^{-16}	-0.365	0.640	0.569	1.032	0.637	0.106
rs10790162	11q23.3	A/G	*BUD13*	0.721	2.901	9.62×10^{-12}	0.144	0.537	0.789	1.037	0.512	0.043
rs964184	11q23.3	G/C	*ZPR1*	0.718	2.869	1.57×10^{-11}	0.140	0.536	0.794	0.990	0.511	0.053
rs6589566	11q23.3	G/A	*ZPR1*	0.721	2.872	1.57×10^{-11}	0.160	0.538	0.766	0.989	0.512	0.053
rs2266788	11q23.3	G/A	*APOA5*	0.721	2.869	1.67×10^{-11}	0.089	0.538	0.869	1.017	0.513	0.047
rs2075290	11q23.3	C/T	*ZPR1*	0.717	2.74	1.09×10^{-10}	0.250	0.536	0.641	0.911	0.512	0.075
rs2367970	11q23.3	G/A	*LOC101929011, BUD13*	0.351	-2.393	2.97×10^{-9}	-0.467	0.502	0.351	-0.975	0.481	0.043
rs1800588	15q21.3	C/T	*LIPC*	0.510	4.447	1.76×10^{-31}	0.482	0.477	0.312	0.395	0.457	0.387
rs1077834	15q21.3	T/C	*LIPC*	0.516	4.437	4.64×10^{-31}	0.360	0.479	0.453	0.343	0.459	0.455

rs1077835	15q21.3	A/G	*LIPC*	0.517	4.433	6.98×10^{-31}	0.361	0.480	0.452	0.342	0.460	0.457
rs261334	15q21.3	G/C	*LIPC*	0.535	-4.171	1.39×10^{-27}	-0.139	0.481	0.773	0.062	0.460	0.893
rs261290	15q21.3	T/C	*AQP9, LIPC*	0.567	-3.663	1.41×10^{-21}	0.040	0.484	0.935	0.122	0.462	0.791
rs2043082	15q21.3	G/A	*AQP9, LIPC*	0.430	3.663	3.18×10^{-21}	-0.047	0.488	0.924	-0.155	0.466	0.739
rs77250403	15q21.3	GA/G	*AQP9, LIPC*	0.434	3.670	5.23×10^{-21}	-0.100	0.493	0.839	-0.171	0.469	0.715
rs261291	15q21.3	T/C	*AQP9, LIPC*	0.526	3.336	4.83×10^{-18}	-0.300	0.484	0.535	-0.422	0.464	0.362
rs1532085	15q21.3	A/G	*AQP9, LIPC*	0.403	-2.914	9.93×10^{-14}	0.022	0.486	0.964	0.576	0.472	0.222
rs10468017	15q21.3	C/T	*AQP9, LIPC*	0.215	3.209	5.10×10^{-12}	-0.591	0.592	0.319	0.098	0.564	0.862
rs4775041	15q21.3	G/C	*AQP9, LIPC*	0.214	3.149	1.30×10^{-11}	-0.599	0.593	0.312	0.102	0.564	0.857
rs8034802	15q21.3	T/A	*LIPC*	0.666	2.552	4.40×10^{-10}	-0.027	0.510	0.957	-0.666	0.491	0.175
rs16940212	15q21.3	G/T	*AQP9, LIPC*	0.316	2.262	3.91×10^{-8}	-0.291	0.522	0.577	0.438	0.501	0.382
rs12148780	15q21.3	A/G	*AQP9, LIPC*	0.153	-2.902	3.95×10^{-8}	1.003	0.665	0.131	0.772	0.640	0.227
rs3764261	16q13	C/A	*HERPUD1, CETP*	0.207	8.863	6.07×10^{-82}	0.817	0.576	0.156	1.357	0.558	0.015
rs183130	16q13	C/T	*HERPUD1, CETP*	0.207	8.858	9.92×10^{-82}	0.783	0.576	0.174	1.338	0.558	0.017
rs247617	16q13	C/A	*HERPUD1, CETP*	0.207	8.848	1.86×10^{-81}	0.791	0.577	0.170	1.336	0.559	0.017
rs17231506	16q13	C/T	*HERPUD1, CETP*	0.209	8.847	2.14×10^{-81}	0.833	0.576	0.148	1.324	0.559	0.018

rs821840	16q13	A/G	*HERPUD1, CETP*	0.207	8.843	4.71×10^{-81}	0.791	0.577	0.171	1.318	0.560	0.019
rs247616	16q13	C/T	*HERPUD1, CETP*	0.207	8.812	6.28×10^{-81}	0.790	0.576	0.170	1.336	0.558	0.017
rs72786786	16q13	G/A	*HERPUD1, CETP*	0.206	8.409	6.40×10^{-58}	0.996	0.655	0.128	1.024	0.633	0.106
rs1532624	16q13	C/A	*CETP*	0.310	6.188	4.31×10^{-52}	0.973	0.512	0.057	0.674	0.487	0.167
rs711752	16q13	G/A	*CETP*	0.401	5.047	2.54×10^{-38}	1.226	0.489	0.012	1.238	0.471	0.009
rs173539	16q13	C/T	*HERPUD1, CETP*	0.316	4.970	4.84×10^{-34}	0.798	0.515	0.122	1.284	0.494	0.009
rs1864163	16q13	G/A	*CETP*	0.102	-7.442	2.75×10^{-32}	0.458	0.785	0.560	-0.922	0.726	0.204
rs7499892	16q13	C/T	*CETP*	0.164	-5.43	1.98×10^{-26}	0.077	0.640	0.904	-0.436	0.608	0.473
rs12708980	16q13	T/G	*CETP*	0.071	-6.418	1.25×10^{-17}	0.478	0.906	0.598	-0.833	0.863	0.335
rs1800775	16q13	C/A	*CETP*	0.552	3.162	1.61×10^{-16}	0.951	0.477	0.046	1.497	0.457	0.001
rs9989419	16q13	A/G	*HERPUD1, CETP*	0.745	2.871	2.81×10^{-8}	0.434	0.657	0.509	0.956	0.636	0.133
rs3786247	18q21.1	T/G	*LIPG*	0.460	3.209	1.02×10^{-16}	0.209	0.490	0.670	0.265	0.465	0.569
rs12970066	18q21.1	C/G	*LIPG*	0.253	2.748	6.04×10^{-10}	0.505	0.557	0.364	-0.093	0.533	0.862
rs35816125	18q21.1	C/G	*LIPG*	0.250	2.645	3.99×10^{-9}	0.334	0.558	0.549	0.172	0.541	0.750
rs429358	19q13.32	T/C	*APOE*	0.100	-3.594	2.98×10^{-8}	0.069	0.802	0.931	-0.276	0.779	0.723

ALT, alternative allele; BMI, body mass index; Coeff., coefficient; GWAS, genome-wide association study; HDL-C, high density lipoprotein cholesterol; REF, referent allele; SE, standard error; SNP, single nucleotide polymorphism.

[a] Adjusted for age, sex, smoking, drinking, daily activity, habitual exercise, egg intake, and BMI. The coefficient value represents change in HDL-C per ALT allele copy (0, 1, 2) for the SNP.
